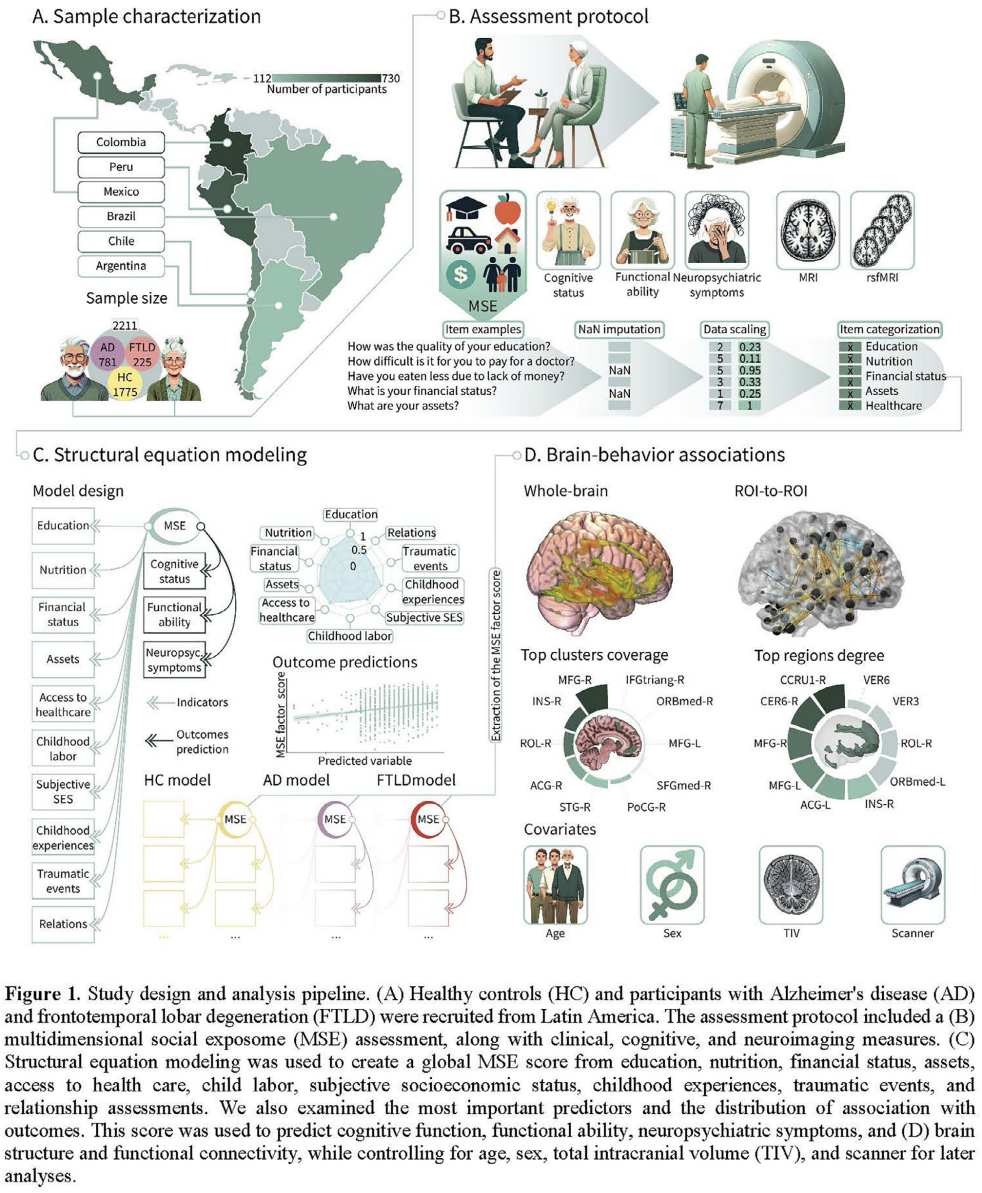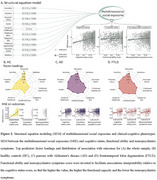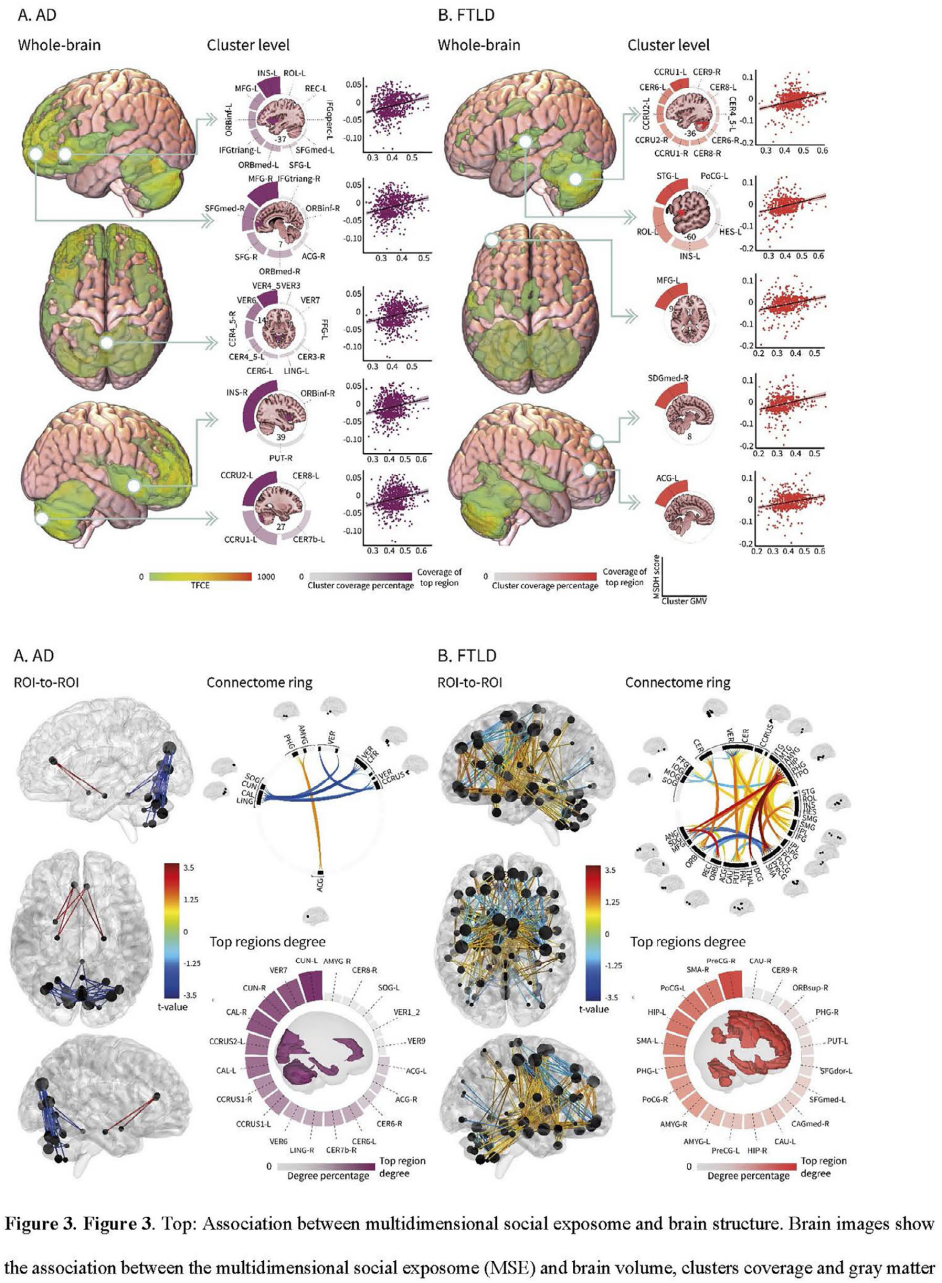# Social exposome and brain health outcomes of dementia across Latin America

**DOI:** 10.1002/alz70856_097406

**Published:** 2025-12-24

**Authors:** Agustin Ibanez, Joaquín Migeot

**Affiliations:** ^1^ Global Brain Health Institute (GBHI), Trinity College Dublin (TCD), Dublin, Dublin, Ireland; ^2^ Latin American Brain Health Institute (BrainLat), Universidad Adolfo Ibañez, Santiago, Chile; ^3^ Global Brain Health Institute (GBHI), University of California San Francisco (UCSF); & Trinity College Dublin, Dublin, Ireland

## Abstract

The social exposome, which involves multidimensional factors associated with economic, health, and social disparities over the life course, can influence the prevalence, progression, and severity of dementia. Such factors are exacerbated in regions like Latin America. The negative impact of low education and socioeconomic status (SES) on dementia is well known. However, the effects of multidimensional lifespan exposome on brain health is unknown. Here, we explore the association between the multidimensional social exposome and brain health outcomes of healthy aging and dementia (Figure 1). An extensive assessment of education, nutrition, financial status, assets, access to healthcare, childhood labor, subjective SES, childhood experiences, traumatic events, and relationship assessments, incorporating different facets of each domain (e.g., parental education, financial stress) were included across the lifespan (from birth to present). We cross‐sectionally assessed 2,211 individuals, comprising healthy controls, persons with Alzheimer's disease (AD), and frontotemporal lobar degeneration (FTLD) from Latin America. Structural equation modeling of cumulative social exposome shown significant associations with poorer cognition in healthy aging, mainly modulated by variables related to different measures of education and socioeconomic status (Figure 2). In people with dementia, the more adverse the social exposome, the lower the cognitive functioning and functional ability, and increased neuropsychiatric symptoms. Nutrition, financial status, subjective SES, and access to healthcare across different life stages were the most critical predictors. Compared to the effect of individual predictors, the cumulative effect of social exposome was more robust in predicting clinical‐cognitive phenotypes across groups. Brain structural and connectivity alterations were associated with more adverse social exposome in dementia‐sensitive and cerebellar regions, particularly frontal and cerebellar hubs in AD, and fronto‐temporo‐limbic and cerebellar regions in FTLD (Figure 3). These findings underscore the extensive impact of multidimensional social exposome on brain health, calling for personalized models of biological‐environmental interactions in underserved populations and tailored prevention efforts.